# Differences in Foliage Affect Performance of the Lappet Moth, *Streblote panda*: Implications for Species Fitness

**DOI:** 10.1673/031.010.14137

**Published:** 2010-10-11

**Authors:** D. Calvo, J.M. Molina

**Affiliations:** ^1^ Departamento de Protección Vegetal, Instituto de Ciencias Agrarias, Centro de Ciencias Medioambientales (Consejo Superior de Investigaciones Cientificas). C/ Serrano 115 dpdo. 28006. Madrid, Spain; ^2^ Crop Protection Area. IFAPA Centro-“Las Torres-Tomejil”. Apdo. Oficial. 41200. Alcalá del Río, Sevilla, Spain

**Keywords:** food utilization, habitat quality, insect-host plant interactions, larvae development, nutritional indices

## Abstract

Implications for adults' fitness through the foliage effects of five different host plants on larval survival and performance of the lappet moth, *Streblote panda* Hübner (Lepidoptera: Lasiocampidae), as well as their effect on species fitness were assayed. Larvae were reared under controlled laboratory conditions on excised foliage. Long-term developmental experiments were done using first instar larvae to adult emergence, and performance experiments were done using fifth instar larvae. Survival, development rates, and food use were measured. Foliar traits analysis indicated that leaves of different host plants varied, significantly affecting larvae performance and adult fitness. *Pistacia lentiscus* L. (Sapindales: Anacardiaceae), *Arbutus unedo* L. (Ericales: Ericaceae), and *Retama sphaerocarpa* (L.) Boiss. (Fabales: Fabaceae) were the most suitable hosts. Larvae fed on *Tamarix gallica* L. (Caryophyllales: Tamaricaceae) and *Spartium junceum* L. (Fabales: Fabaceae) showed the lowest survival, rates of development and pupal and adult weight. In general, *S. panda* showed a relatively high capacity to buffer low food quality, by reducing developmental rates and larvae development thereby reaching the minimum pupal weight that ensures adult survival. Less suitable plants seem to have indirect effects on adult fitness, producing smaller adults that could disperse to other habitats.

## Introduction

Habitat quality, corresponding to differences in biotics or abiotics factors that may affect in different ways herbivore performance, reproduction success, or abundance, are major themes of research. Plant phenology or foliar chemistry (Schultz et al. 1982; Hunter 1992), mainly expressed as variations in foliar carbohydrate levels (Valentine et al. 1983), nitrogen (Mattson 1980; Lovett et al. 1998), or secondary compounds (Rossiter et al. 1988; Bourchier and Nealis 1993), together with abiotic factors such as temperature or photoperiod, can modify species performance in diverse ways. The ability of the species to overcome these factors by means of adaptations in its physiology and behavior will determine its biological success.

The lappet moth, *Streblote panda* Hübner, (Lepidoptera: Lasiocampidae), lives in low and littoral areas of the Iberian Peninsula and North Africa from Egypt to the Atlantic coast of Morocco. Across this range, *S. panda* uses taxonomically diverse species as host plants; some of them are of forest, floricultural, or horticultural interest (Zhang 1994; Molina 1998; Calvo 2004). Previous work has measured performance parameters of *S. panda* on several host plants, including some commercial varieties of blueberries in southwestern Spain, where this species is able to completely defoliate two year old plants (Calvo and Molina 2004 a, b).

The population dynamics of this species are greatly influenced by host quality (Calvo and Molina 2005b). The number of generations is normally difficult to determine because of the presence of asynchronous individuals. These asynchronous individuals are generally produced as a consequence of the combination of biotic and abiotic factors resulting in alterations of the normal pattern of resource allocation that produce an alteration of larval performance. *S. panda* is generally bivoltine, with one generation in early spring and a second in summer. In southwestern Spain, the second generation cannot complete development and offspring usually hibernate as mature larvae or pupae (Calvo and Molina 2005a). Because adults do not feed, the quantity and quality of food ingested as larvae will strongly influence the amount of reserves stored in the abdominal fat body that will be allocated for reproduction and dispersion during the adult phase (Calvo and Molina 2005c).

For most of the important insect pests, the interactions between insects and their common crop hosts are well-known. Conversely, understanding of the interactions between insect pests and naturally occurring non-crop host plants are poorly understood. Naturally occurring host plants can be a reservoir where insects maintain healthy populations, and these can be the origin of colonizing individuals towards crops. Studies of the interaction between insect pests and non-crop host plants will contribute to understanding how the insect regulates its physiology and behavior in order to maintain healthy metapopulations. The main objective of this study was to evaluate the consequences of host plant use at the level of host plant species for herbivore fitness.

## Material and Methods

### Plants

Host plants used in this study were selected on the basis of previous sampling records of wild populations of *S. panda*. Bibliographic records and observations of *S. panda* in the field seem to show a differential use of host plants throughout the year as well as in its geographic range of distribution. These plants were Mastic, *Pistacea lentiscus L*. (Sapindales: Anacardiaceae), strawberry tree, *Arbutus unedo L*. (Ericales: Ericaceae), yellow broom, *Retama sphaerocarpa (L.) Boiss*. (Fabales: Fabaceae), Spanish broom, *Spartium junceum L*. (Fabales: Fabaceae), and French Tamarisk, *Tamarix gallica L*. (Caryophyllales: Tamaricaceae). French Tamarisk may also be of special interest due to its invasive status in some parts of the world (Bowman 1990).

Plant material of all host plants used was collected from Seville (Andalusia, Spain), where they have been used in public gardens, road verges, and edges of agricultural fields. The plant material was collected at different periods of time based on records of *S.panda* in field samplings. *P. lentiscus, A. unedo*, and *S. junceum* were collected from early spring to early summer; *T. gallica* was collected from late spring to late summer; and *R. sphaerocarpa* was collected from late summer to winter. Twigs of approximately 25 cm in length were selected from each plant species. Tips of the twigs were removed so that only completely developed leaves were offered. To minimize stress or induction of any defensive responses, foliage was cut from different individual shrubs at each feeding date. Leaves were sampled weekly over the whole experimental period, beginning when the laboratory feeding experiments started and finishing when larvae had completed their development. For each plant species, some of the leaves collected were used for chemical determinations.

### Insects

Larvae of *S. panda* used in this study were obtained from a colony maintained in our laboratory at IFAPA Centro Las Torres-Tomejil (Alcalá del Río, Seville, Spain). The colony was established from mature larvae collected in Moguer (Huelva, Spain). In order to avoid endogamy and any interference due to host preference, from time to time new wild specimens obtained from different host plants and locations were introduced in the colony. The eggs used in the experiments were isolated within the first 24 h after oviposition. Females and eggs did not have any contact with plants. All larvae used in the experiments were randomly chosen from the stock culture.

### Larval growth and performance experiments

Bioassays were conducted in order to determine the effect of nutritional changes on larval mortality, growth, and performance of *S. panda*. Using leaves of the five species of selected host plants long-term developmental experiments were done using first instar larvae to adult emergence, and performance experiments were done using fifth instar larvae. In order to restrict the factors affecting performance, abiotic conditions were maintained constant in all experiments (25 ± I° C, 70 ± 5% RH, and 16:8 L:D photoperiod).

To evaluate the effects of host plant on development and growth of the insect during the entire larval feeding and pupal stages, 15 neonate larvae per host plant assayed were isolated in 500 ml plastic rearing containers lined with moistened filter paper and maintained in a growth chamber. Larvae were kept together in the same rearing cage from hatching until the third instar when individual larvae were placed in separate containers. Larvae were observed daily and molting, survival, and weight were recorded from the 3^rd^ instar until the adult eclosed. Every second day new food was supplied, and remaining leaves and frass were removed.

For each individual the following traits were recorded or measured: Developmental time from hatching to pupation and adult eclosion, growth rate, pupal and adult weight and adult wing length. Larval duration was calculated as the total time from egg hatching until pupation. The individual growth rate was calculated according to Gotthard et al. (1994): growth rate = [In (pupal weight) = In (hatching weight)]/larval time. This formula indicates the mean weight gain per day. Each pupa was weighed within 24 h after pupation and was kept in individual containers until adult emergence. Pupal duration was calculated as the time between pupation and adult emergence. Adults were weighed within 24 h after emergence and the length of the right forewing was measured.

Fifth instar larval performance experiments were conducted to evaluate the host plant effects on growth rates, food consumption, and efficiency of food processing (Scriber and Slansky 1981). Larvae were fed every two days ad libitum until they moulted to the fifth instar. From this point, each larvae was weighed daily along with food remains, frass produced, and new leaves provided. Ingestion, growth, and egestion were directly measured; metabolism was determined by difference. Nutritional parameters were calculated in order to assess insect growth and consumption, and food utilization efficiencies were estimated by standard gravimetric techniques (Scriber and Slansky 1981; Smith et al. 1994). Indices were calculated on a dry weight basis. They were: approximate digestibility, AD = 100*(ingestion-frass)/ingestion, efficiency of conversion of digested food, ECD = 100*biomass gained/(ingestion-frass), efficiency of conversion of ingested food, ECI = 100*(biomass gained/ingestion), relative growth rate, RGR = biomass gained/We/day, and relative consumption rate, RCR = ingestion/We/day (Scriber and Slansky 1981). Relative rates were based on the mean exponential larval dry weight (We) according with the expression: We = (Wf-Wi)/ln(Wf/Wi) where Wf and Wi are final and initial dry weights, respectively (Gordon 1968). Calculations for nitrogen utilization included: relative nitrogen accumulation rate, RNAR = nitrogen gained/We/day, relative nitrogen consumption rate, RNCR = nitrogen ingested/We/day, and nitrogen utilization efficiency, NUE = 100*nitrogen gained/(N ingested-N excreted) (Scriber 1977).

Dry weight of experimental larvae was estimated by multiplying their fresh weight by the mean dry weight percentage, determined from lots of ten larvae reared on each host plant in the same conditions as the experimental specimens and randomly sacrificed at intervals during the experience. Dry weight of food offered was estimated from the mean dry weight:fresh weight ratio of control leaves, cut at the same time and from the same host plant as the leaves used as food. Larvae and food were dried in an oven at 60° C for 48h.

### Foliar chemistry of plant material

Basic analysis of plant components and proportions were done using plant material previously dried at 60° C for 48 h and ground to a fine powder in a laboratory mill. Total nitrogen content was determined by the Kjeldahl technique (Allen 1989) and expressed as percentage of the dry weight. Percentage of leaf water was determined gravimetrically, by the ratio of leaf dry to fresh mass. Levels of total phenolics were determined using 80 mg of freeze-dried leaves following the extraction method of Stadler-Martin and Martin (1982), and the Folin-Ciocalteau colorimetric method for concentration determination (Allen 1989). Total carbohydrates in plant material were determined by the anthrone method, following the extraction protocol contained in Allen (1989).

### Statistical analysis

Foliar carbohydrates, nitrogen, water content and phenols were subjected to analysis of variance (ANOVA), with host plant species as the independent variable. Percentages of larval survival on respective plants were analyzed using a non-parametric Kruskall-Wallis test for more than two independent samples. Paired comparisons (Mann-Whitney U-test) were performed in order to determine the differences in survival between and within host plants. The significance level chosen for both non-parametric tests was 0.05.

A multivariate analysis of covariance (MANCOVA) was applied to fifth instar performance data using host plant and adult sex as fixed factors, and the initial weight of larvae as the covariate to correct the effect of variation in initial mass on intake and growth. For the rest of the parameters analyzed an analysis of variance was applied. Significance level chosen for tests was 0.05. Treatments means from significant ANOVA/MANCOVA tests were separated by Newman-Keuls multiple range tests. Percentage data were transformed by angular transformation: x'=arcsine (√x) and the logarithmic transformation: x'=log (x+1) was used for remaining data (Dent and Walton 1997).

**Table 1.**
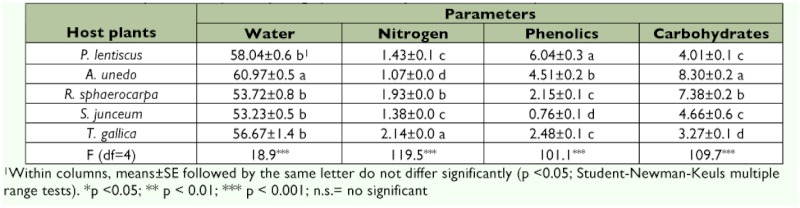
Nutritional parameters (% of dry weight) of five host plant used to rear *S. panda*.

Pearson's correlation coefficient was used to detect associations between plant nutritional factors and larval performance (Dent and Walton 1997). All statistical analyses were performed using SPSS statistic v. 17 software.

## Results

### Host plant quality

Analysis of foliar components indicated that leaves of the different host plants varied significantly (Table 1). The different host plant species analyzed, and the time when they were collected, determined these differences. Higher water content was associated with higher total phenolics content of plant leaves (r = 0.36, n = 48, p <0.05). The rest of the nutrients analyzed showed higher variability between plants. *T. gallica* leaves had the highest nitrogen content, but conversely had low total phenolics and carbohydrates content. On the other hand, plants like *P. lentiscus* and *A. unedo* had lower nitrogen levels in combination of higher total phenolics content. *R. sphaerocarpa* and *A. unedo* had the highest carbohydrate content. *S. junceum* had low nitrogen and the lowest phenolics content.

### Larval growth and performance experiments

A clear effect of host plant nutritional quality on *S. panda* larvae growth and performance was found. No significant effects on larval survival were detected until the fifth instar. All experimental larvae fed with *P. lentiscus* and *A. unedo* were able to complete their development. Larvae fed on *R. sphaerocarpa* recorded a low survival rate, especially during the adult phase (Mann-Whitney *U*-test for adult survival, p = 0.035). In the case of *S. junceum* low survival was detected for the last instars, pupal and adult stages (Mann-Whitney *U*-test, p_5th larvae_ = 0.317, p_6th larvae_ = 0.317, p_pupae_ = 0.000, p_adult_ = 0.000). Larvae survival fed on *T. gallica* decreased during larvae development. Only 40% of the initial larvae fed on this plant were able to pupate (Mann-Whitney U-test, p_5th larvae_ = 0.016, p_6th larvae_ = 0.007, p_pupae_ = 0.000, p_adult_ = 0.000) (Table 2).

**Figure 1.**
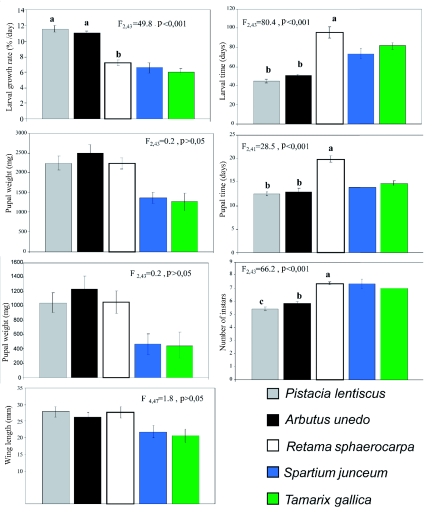
Performance of *Streblote panda* on five host plants species, p-values represent the significance of analysis of variance. Bars with the same letter do not differ significantly (p <0.05; Student-Newman-Keuls multiple range tests). Vertical lines indicate ± SE N = 15. Data from *S. juceum* and *T. gallica* have been excluded from the analysis. High quality figures are available online.

Species performance, as estimated from the long-term study, depended on the plant on which the larvae developed; each plant showed three different tendencies (Figure 1). Larvae reared on *P. lentiscus* and *A. unedo* showed a tendency characterized by fast larval growth rates that resulted in low developmental times and instar number, and high pupae and adult weight. The opposite tendency occurred in the case of the larvae reared on *S. junceum* and *T. gallica*. On these host plants, larval growth rate was low resulting in longer larval developmental times, and longer pupal developmental time that resulted in significantly reduced pupal and adult weights (Figure 1). Larvae fed on *R. sphaerocarpa* showed the third tendency, with a quite different performance. Low larval growth rates were associated with significantly higher larval developmental time, but opposite to the larvae fed on *T. gallica* or *S. junceum*, pupal and adult weights were not significantly different from *A. unedo* or *P. lentiscus* larvae. No sex related differences were detected in pupal development time. Only one male and one female were obtained for *S. junceum*; and these data were excluded from the analysis (F_4, 37_ = 1.5, p >0.1). Females were heavier that males (F_4, 37_ =2.8, p <0.01) in all the host plants analyzed (Table 3). No significant differences were detected in wing length between the different plants (see also Figure 1).

**Table 2.**
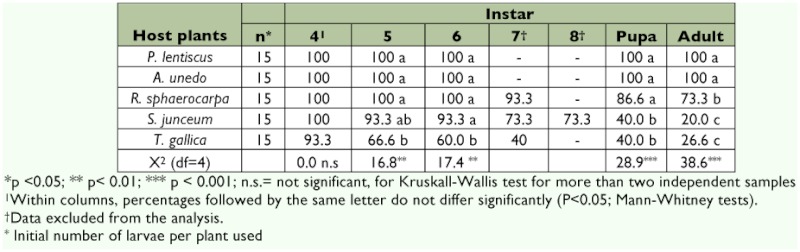
Percentage of survival of *S. panda* larvae reared on different host plants along development. Survival from the first to the third stadia was in all the cases of 100%.

Host plant was the main factor that influenced fifth instar development (F_96, 152_ = 12.9, p <0.001). Some of the individuals died before they reached the fourth instar which resulted in lower sample sizes, especially in the case of larvae reared on *S. junceum* with only nine larvae reaching the fourth instar (Table 4). Larvae fed on *T. gallica* and *R. sphaerocarpa* showed lower weight gains with longer instar duration than those fed on the other plants assayed. Conversely, larvae fed on *S. junceum* showed the lowest weight gain, but the shortest instar duration (Table 4). Again, sex did not significantly affect fifth instar duration (F_4, 39_ = 0.4, p >0.1) or weight gain (F_4, 39_ = 0.6, p >0.1).

Relative rates of consumption and growth showed fewer variation between host plants. Lower RGR was inversely correlated with the increment of instars duration (r _RGR-INSTAR_ = 0.64, n = 48, p <0.001). Higher values of RCR produced a decrease of both efficiencies values (r _RCR-ECI_ = -0.71, n = 48, p <0.001; r _RCR-ECD_ = -0.57, n = 48, p <0.001).

The main differences between plants were detected on food utilization efficiencies (ECI, ECD, and AD) where the more suitable plants showed the highest values (Table 4). Larvae fed on *S. junceum* showed a high efficiency in assimilation of the food ingested with low growth rates. This indicated that the larvae were able to efficiently assimilate most of the food consumed. Low growth rates were a consequence of longer periods of time between meals, indicating more time spent in the digestion progress.

A decrease in the efficiency of conversion of ingested and digested food was correlated with lower total phenolics or carbohydrates levels (r _phenols-ECI_ = -0.35, n = 48, p <0.05 and r _phenols-ECD_ = -0.53, n = 48, p <0.01; r _carbohydrates-ECI_ = -0.38, n = 48, p >0.05 and r _carbohydrates-ECD_ = -0.54, n = 48, p <0.01). Conversely, the increment of these two compounds increased the efficiency of digestibility (AD) (r _phenols-AD_ = 0.57, n = 48, p <0.001 and r carbohydrates-AD = 0.51, n = 48, p <0.01).

**Table 3.**
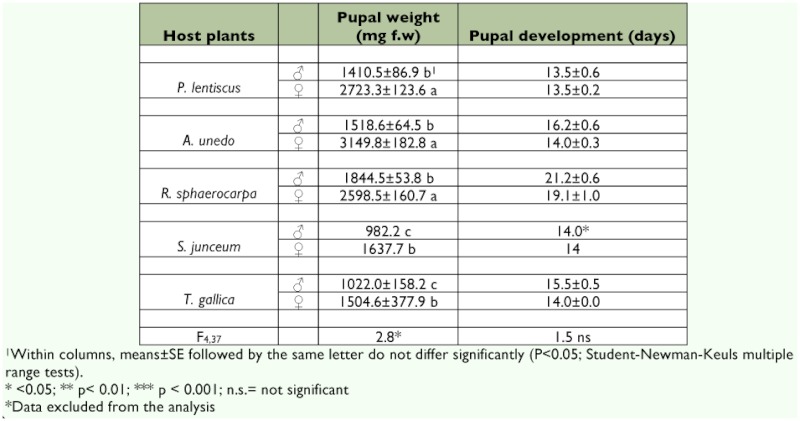
Pupal weight and pupal development time of males and females of *S. panda* fed on five different host plants.

**Table 4.**
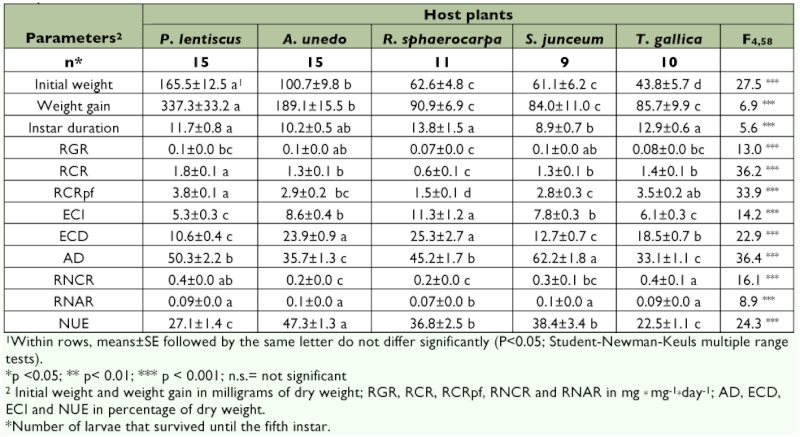
Growth parameters, nutritional indices and utilization of nitrogen of *S. panda* fifth instars larvae fed on five different host plants.

The relative amount of nitrogen resulted in large differences in RNCR, with larvae fed on *R. sphaerocarpa* showing the lowest values (Table 4). However, RNAR showed little variation among plants; only mean values of this parameter for larvae fed on *R. sphaerocarpa* (the host plant with the second highest value of total nitrogen content) was significantly different (Table 4). It is remarkable that the high NUE was obtained for larvae fed on *A. unedo* and the lowest were recorded for larvae fed on *T. gallica* (Table 3). Higher RCR also resulted in lower nitrogen efficiency (r RCR-NUE = -0.45, n = 48, p <0.001).

## Discussion

The general importance of behavioral, physiological, and ecological adaptation of herbivores to their host-plants is recognized as a main theme of coevolution (Scriber and Slansky 1981). Insect distribution in a subset of the available plant hosts presumably reflects the interaction of consumers and plant characteristics, although the predators of consumers and competition from other herbivores might also play an important role (Jervis et. al. 2007; Ricklefs 2008). The success of polyphagous species, such as *S. panda*, on a particular plant depends on their physiological ability to buffer changes in food composition, either due to alterations in the nutritional parameters or to the presence of secondary compounds that many plants produce that inhibit herbivore feeding because they are unpalatable, toxic, or both (Bernays and Chapman 1987; Chapman and Sword 1994; Cambini and Magnoler 1997).

Plants with low amounts of soluble carbohydrates, low nitrogen, and relatively high levels of polyphenols and potassium can have the largest number of species of Lepidoptera consuming them in spite of relatively low foliage “quality” (Ricklefs 2008). Protein and carbohydrate ratios play different physiological roles in insect development (Raubenheimer and Simpson 1997). Two of the plants tested that showed the greatest physiological effects on larval development were *T. gallica* and *S. junceum*. Higher nitrogen content relative to carbohydrate in the case of *T. gallica* may be responsible for the higher mortality, longer developmental times, and lower pupal mass recorded on this plant. Studies on *Spodoptera littoralis* also showed a decrease in pupal mass on a diet with a high protein to carbohydrate ratio (Lee et al. 2002). Conversely, the ratios between these two nutrients in *S. junceum* are similar to the ratio in the other plants tested. Food palatability can change as a function of nutritional components or structural components. Structural components, like trichomes, fiber content, or leaf pilosity have been previously described as influencing insect development (Angelini et al. 2000; Sharma et al. 2009). *S. junceum* has been used traditionally as a fiber crop due to its high fiber content (Angelini et. al. 2000). Fiber content could be increasing in the summer, making it more difficult for the larvae to digest the leaves and extract the nutrients necessary for development, and expending more time between meals which decreases their consumption rates.

*S. panda* larvae showed lower growth rates as well as a reduction on conversion efficiencies on some plants. These facts represent a possible complication in the nutrient digestion and absorption, which result in an increase in larvae development and instar numbers. Supernumerary larval instars are directly related to low foliage quality (Kamata and Igarashi 1995; Nijhout 2003). On three of the five plants tested- *T. gallica, S. junceum*, and *R. sphaerocarpa*- supernumerary instars were detected. An increase in the number of larval instars and developmental time on these plants may indicate the existence of a mass threshold for pupation. If the threshold is not reached, then the larva will continue the moulting cycle until a critical mass is attained (Nijhout 2003).

Besides the capacity of larvae to buffer or regulate host plant quality, the female oviposition choices between the potential host plants can also contribute to the success of larval performance (Janz and Nylin 1997). However, empirical evidence often shows no correlation or ambiguous correlations between female oviposition preference and larval performance (Agosta 2008). After larval eclosion, in addition to other variables, the quality of the food will influence the rest of development. Previous studies have recorded that the first two larval instars of the forest tent caterpillar are the critical period in which the larvae grew and developed more slowly (Jones and Despland 2006). Suboptimal foliage quality did not result in an increase in mortality in first two instars of *S. panda* larvae, but an increase in instar developmental times and in mortality in late instars were detected. In general, the nutritional quality of shrubs and trees decreases with leaf maturity. The results of this study showed that differences on food quality caused wide variations of *S. panda* larval development, which lasts between 5 to 21 weeks depending on the host plant under the conditions of the same temperature and photoperiod (Figure 1). Longer periods of time on the plant imply that early- and late-instar larvae forage on foods where the chemical and physical composition are different, which could have a consequence on growth and development affecting their survival, larval instar numbers, nutritional parameters, or pupal weight (Mattson and Scriber 1987). Indeed, as has been mentioned before, larvae are able to protract their development, as was seen on *R. sphaerocarpa* plants. Feeding on inadequate or poor quality foliage has also a direct effect on adult size, female fecundity, and dispersal ability (Kamata and Igarashi 1995; Braschler and Hill 2007), which will have a lasting influence on the maintenance of healthy populations.

The inability of *S. panda* adults to feed forces the larvae to obtain all the resources needed to maximize the reproductive success of the species. Better larvae development will produce bigger and healthier adults that will increase species fitness (Calvo and Molina 2005c). In that sense, the larval food plant may influence adult dispersal in several ways. Suboptimal or inadequate food may limit energy reserves in adults as well as change the allometric relationship between adult body mass and wing length; reflecting either attempts to conserve reproductive potential or possible changes in wing loading (Boggs and Freeman 2005; Braschler and Hill 2007). The fitness of a female is determined by the number of viable offspring that she can produce, a factor strongly dependent upon her body size (Calvo and Molina 2005c). Moths developing on *T. gallica* and *S. junceum* attained lower body weight and lower wing loading and, as consequence, lower total egg load. It is expected that these adults that came from larvae developed on low quality hosts, have higher tendency to disperse, looking for a more suitable host plant. The output change in the case of *R. spaherocarpa*, where the adults produced had a medium size with no change in wing loading in comparison to the most suitable host. From field data, it is known that *R. sphaerocarpa* is able to produce hibernating larvae that may be viewed as a reservoir of healthy individuals that could disperse to other plants as the season progresses.

Current literature shows that parasitism or predation and habitat quality can have an influence on the presence of butterflies and moths in different habitats. This can explain much of the patch occupancy pattern in some species (Cassel-Lundhagen et al. 2008). Predation-induced phenotypic plasticity is widespread in nature and includes variation in life history, morphology, and behavior (Bernard 2004). Mimetic coloration, long periods of time between meals, or urticating retractable organs on the metathorax are some of the adaptations developed by this species to avoid parasitism or predation (Berger et al. 2006; Calvo and Molina 2008). Lower quality habitat, understood as poor food quality as in the case of *T. gallica* or *S. junceum*, should increase larvae developmental times in order to increase their final size and produce adults as large as possible and with as high an egg load as possible. Changes in the food quality may increase species mortality and parasitisation rate. A tachinid parasitoid specific to this species has been recorded preferentially on larvae fed in *T. gallica* during field samplings (Calvo and Molina 2002). As consequence, it is expected that the species may show a lower occupancy in these habitats together with smaller adults that should disperse away from the originating population.
